# Body part-centered and full body-centered peripersonal space representations

**DOI:** 10.1038/srep18603

**Published:** 2015-12-22

**Authors:** Andrea Serino, Jean-Paul Noel, Giulia Galli, Elisa Canzoneri, Patrick Marmaroli, Hervé Lissek, Olaf Blanke

**Affiliations:** 1 Laboratory of Cognitive Neuroscience, Brain Mind Institute, School of Life Sciences, Ecole Polytechnique Fédérale de Lausanne, Lausanne, Switzerland; 2 Center for Neuroprosthetics, School of Life Sciences, Ecole Polytechnique Fédérale de Lausanne, Lausanne, Switzerland; 3 Department of Psychology, Sapienza University, Rome, Italy; 4 Laboratory of Electromagnectics and Acoustics, Institute of Electrical Engineering, School of Engineering, Ecole Polytechnique Federale de Lausanne, Lausanne, Switzerland; 5 Department of Neurology, University Hospital, Geneva, Switzerland

## Abstract

Dedicated neural systems represent the space surrounding the body, termed Peripersonal space (PPS), by integrating visual or auditory stimuli occurring near the body with somatosensory information. As a behavioral proxy to PPS, we measured participants’ reaction time to tactile stimulation while task-irrelevant auditory or visual stimuli were presented at different distances from their body. In 7 experiments we delineated the critical distance at which auditory or visual stimuli boosted tactile processing on the hand, face, and trunk as a proxy of the PPS extension. Three main findings were obtained. First, the size of PPS varied according to the stimulated body part, being progressively bigger for the hand, then face, and largest for the trunk. Second, while approaching stimuli always modulated tactile processing in a space-dependent manner, receding stimuli did so only for the hand. Finally, the extension of PPS around the hand and the face varied according to their relative positioning and stimuli congruency, whereas the trunk PPS was constant. These results suggest that at least three body-part specific PPS representations exist, differing in extension and directional tuning. These distinct PPS representations, however, are not fully independent from each other, but referenced to the common reference frame of the trunk.

Neurophysiological studies in monkeys described a pool of multisensory neurons-mainly in ventral premotor cortex, ventral intraparietal area, area 7, and putamen – dedicated to represent the space surrounding the body, termed Peripersonal space (PPS)^1^. Typically these neurons have a tactile receptive field (RF) centered on a specific body part (hand, arm, face, trunk or shoulder, with some neurons having RFs covering even the entire body) and a visual and/or an auditory RF that overlaps spatially with the tactile RF and extends in depth for a distance ranging from 5 to 100 cm from the body^2^,^3^,^4^.

Neuropsychological studies in patients^5^ and behavioral studies in healthy participants^6^ provided first evidence for the existence of a PPS system in the human brain. These studies showed that tactile perception is more strongly modulated by visual or auditory stimuli^7^,^8^ when these are presented close, as compared to far, from the body. Neuroimaging studies^9^,^10^ associated these effects with neuronal processing in fronto-parietal brain regions, homologues to the regions hosting PPS neurons in non-human primates.

Whereas, the properties of PPS neurons in monkeys in terms of receptive field size, response preferences, and spatial frames of reference have been extensively described, knowledge about the characteristics of PPS representation in humans is mostly limited to the evidence of stronger multisensory interaction near the hand or the face. Therefore, it is still debated to what degree PPS representation in the human brain is based on the same properties as those implemented in monkey PPS neurons^11^,^12^.

The aim of the present study is to measure behavioral responses in humans reflecting the concept and the properties of RFs in primate PPS neurons. To this aim, we applied a recently developed paradigm in which participants are requested to respond as fast as possible to tactile stimulation administered on a part of their body, while task-irrelevant approaching or receding external cues (auditory or visual stimuli) are presented^13^,^14^,^15^,^16^. On each trial, the tactile stimulus is administered at a different temporal delay from the cue onset and thus touch is processed when the cues are perceived at a different distance from the participant’s body. In this way, we determine the critical distance at which an external stimulus affects tactile processing. This point can be considered as the boundary of PPS representation in humans.

In a series of 7 experiments (164 participants), we applied this method to study the properties of the human PPS. By administering tactile stimuli to different body parts, we firstly compared the extension of PPS representation around the hand, face, and trunk. Then, we studied the relationships between discrete PPS representations around different body parts, by either varying the relative position of the arm and the trunk, while sounds approached the hand or the trunk (Experiments 1–5), or by varying the congruency between tactile stimulation on the face and trunk, while auditory or visual stimuli either congruently approached the stimulated body part or incongruently the other body part (Experiments 6–7). Taken together, our findings suggest that at least three body-part specific PPS representations exist (differing in extension and directional tuning), which are all referenced to the more common reference frame of the trunk.

## Results

### Section 1. The relationship between Peri-trunk and Peri-hand PPS

#### Experiment 1. Peri-trunk PPS

**Figure 1:**
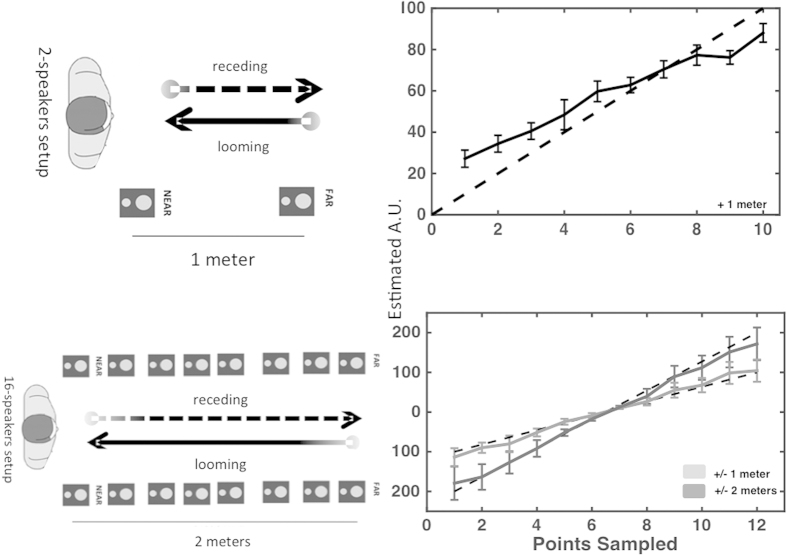
Experimental Setup. Upper Left Panel; illustration of the 2-speaker setup utilized in Experiment 1, 3, 4, and 5. Auditory looming and receding sounds were simulated by modulating the intensity emitted by a near (5 cm) and a far (100 cm) speaker. Upper Right Panel: Estimate of distance (in an arbitrary unit–A.U) from 0 (near) to 100 (far) as a function of the moment when participants received a tactile stimulation, while a looming sound, originating from the far loudspeaker and illusorily moving towards the near loudspeaker, was presented. Error bars indicate +/−1 S.E.M. Lower Left Panel: illustration of the 16-speaker setup utilized in Experiment 2 and 6. Looming and receding sounds were simulated by placing participants between two arrays of 8 speakers (2 meters of longitudinal distance, and 50 cm between participant midline and each array of speakers in the horizontal plane), and modulating the intensity of the sound produced by each speaker as a function of time. Lower Right Panel: Estimate of sound distance as a function of point in space sampled for the 16-speaker setup. Participants estimated sound distance for sounds originating 1 meter in front (positive x-value) and terminating 1 meter behind (negative x-value) in light gray, and for sounds originating 2 meters in front and terminating 2 meters behind the subjects in dark gray. Error bars indicate +/−1 S.E.M. (Figure drawn by JPN and EC)

The first experiment aimed at investigating PPS representation around the trunk. We tested whether an auditory stimulus interacted more strongly with a tactile stimulation on the chest when presented within a specific spatial range from the trunk. We determined the farthest point in space in which a sound significantly speeded up tactile RT as compared to a baseline unimodal tactile RT, and this point was taken as a proxy for the boundary of peri-trunk PPS. To this aim, participants were requested to respond as fast as possible to a tactile target administered to their trunk (chest, at sternum level), while task-irrelevant sounds either approached or receded from their trunk. On each trial, the tactile target was presented at one out of six possible delays from sound onset, i.e. when sounds were perceived at one out of six possible distances (D1, D2, D3, D4, D5, and D6) between 5 and 100 cm from the body (D1 through D6 respectively corresponding to 5, 24, 43, 62, 81, and 100 cm). Baseline trials (in which no auditory stimuli was provided) and catch trials (in which no tactile stimulation was given) were also acquired (see Fig. 1; see also Methods section for further details). Averaged tactile RTs for each of the six possible distances were corrected for RT to unimodal tactile stimuli. We firstly searched for a difference in multisensory RT at consecutive distances, to show a spatially dependent modulation of tactile processing and then we compared corrected RT at each distance to baseline (by definition, zero) in order to identify the farthest point at which a multisensory facilitation occurred as the boundary of peri-trunk representation (all subsequent studies follow the same logic).

**Figure 2:**
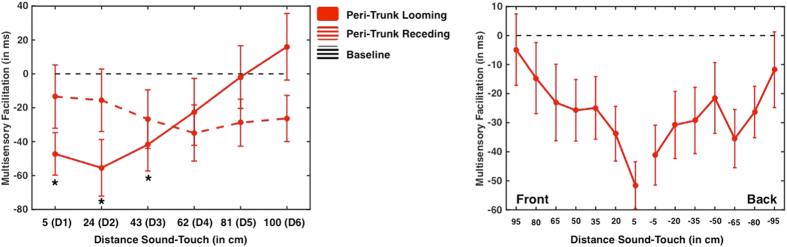
Peri-Trunk Space. Upper Panel-Experiment 1; Multisensory facilitation (in ms; negative values indicate multimodal audio-tactile RT < unimodal baseline tactile RT) as a function of sound distance at the moment of tactile stimulation (D1 indicates the smallest distance, while D6 indicates the largest distance). RT facilitations due to looming sounds are illustrated in solid red, while responses to receding sounds are portrayed in dashed red. The black dashed horizontal line indicates baseline, i.e., RT to the fastest unimodal tactile stimulation, and *indicate a significant facilitation effect with respect to baseline (p < 0.05 Bonferroni-corrected Error bars indicate +/−1 S.E.M.). Lower Panel-Experiment 2; Multisensory facilitation (in ms; negative values indicate multimodal audio-tactile RT <unimodal baseline tactile trials) as a function of sound distance at the moment of tactile stimulation (D1 closest; D7 furthest), in the front (left) and back (right) space. The black horizontal dashed line indicates the fastest baseline RT to tactile stimulation alone. Error bars indicate +/−1 S.E.M.

Baseline-corrected RTs were submitted to a 2 (Sound Direction: Looming vs. Receding) × 6 (Sound Distance: D1 through D6) Within-Subjects ANOVA. As illustrated in Fig. 2A, findings showed a significant Sound Direction × Sound Distance interaction (F(5, 65) = 18.743, p < 0.001, partial η^2^ = 0.590). In order to identify the source of the interaction, two separate ANOVAs were conducted for each sound direction separately, with Sound Distance as a main Factor. A significant effect of Sound Distance was found for Looming sounds (F(5, 65) = 38.453, p < 0.001, partial η^2^ = 0.583). Bonferroni-corrected multiple comparisons revealed that RTs to tactile targets for sounds at D1 (5 cm), D2 (24 cm), and D3 (43 cm) were all significantly (p < 0.05, corrected) faster than the unimodal tactile baseline, suggesting that the boundary of PPS was located between D3 (43 cm) and D4 (62 cm). In contrast, no Sound Distance effect was found for the receding sounds (F(5, 65) = 0.604, p = 0.314), indicating that no spatially dependent modulation of tactile RT due to sound distance was found for the peri-trunk PPS when receding sounds were presented (all p > 0.12).

Results from Experiment 1 show that looming sounds modulated tactile processing at several test positions, namely from D1 (5 cm) to D3 (43 cm), and that this modulations was a function of the distance of the sound from the body. Moreover, the effect was selective for looming sounds and absent for receding sounds.

#### Experiment 2. Front and back peri-trunk PPS

Most previous studies on PPS representation focused on visuo-tactile stimulation and therefore tested uniquely the front space. Hence, evidence concerning the existence and the nature of the back PPS is poor. In Experiment 2, we took advantage of our audio-tactile stimulation paradigm in order to test whether the representation of the peri-trunk space is similar for the front and the back space. To this aim, we applied tactile stimulation to the trunk, at the same time on the sternum or at an equivalent elevation on the back. Concurrently, we presented dynamic sounds, starting from a far position (at 1 m), either in the participant’s front or back space, and terminating at a far location at the same distance, but in the opposite space, namely, back and front. This way, sounds were looming in the first part of their trajectory and then receding once they passed the participants’ body. On each trial, tactile stimulation was given at one of 14 delays (5, 20, 35, 50, 65, 80, and 95 cm, both in the front and in the back).

Baseline-corrected RTs were submitted to a 2 (Mapping Space: Front vs. Back) × 2 (Sound Direction: Looming vs. Receding) × 7 (Sound Distances) Within-subjects ANOVA. Analysis exposed a significant Sound Direction × Sound Distance interaction (F(5, 75) = 3.779, p < 0.01, partial η^2^ = 0.201). Individual 2 (Mapping Space) × 7 (Sound Distances) Within-Subjects ANOVAs were ran for Looming and Receding sounds. As illustrated in Fig. 2B, the looming sounds did elicit a significant Sound Distance effect (F(5, 75) = 6.348, p < 0.001, partial η^2^ = 0.297). There was no main effect for Mapping Space (F(1, 15) = 3.420, p = 0.084, η^2^ = 0.101), nor a significant Mapping Space × Sound Distance interaction (F(5, 75) = 1.062, p = 0.388). In order to study the main effect of Sound Distance, we averaged RT at the different distances for the front and the back space. Bonferroni-corrected comparisons to baseline showed that RT for all points besides D7 (95 cm) were significantly faster than that from the unimodal tactile condition (all p < 0.05, corrected). No significant differences emerged concerning the extent of the PPS representation in the front versus back space. As found in Experiment 1, in the case of receding sounds, no significant main effect of Distance (F(5, 75) < 1, ns), Mapping Space, or interaction was found, thus confirming that sounds receding from the trunk are not mapped into the representation of the peri-trunk PPS.

We note that the PPS boundary was identified at a farther distance in Experiment 2 (between 95 and 80 cm) than in Experiment 1 (between 42 and 63 cm). This difference might be due to a number of (non-mutually exclusive) different methodological factors. There were differences in the trajectory, the source, and the velocity of sounds used in the two experiments, and the last factor in particular might explain the different PPS boundaries. The sounds approached participants at a faster speed in Experiment 2 (35 m/s) than in Experiment 1 (22 cm/s). Previous neurophysiological recordings in monkeys^3^ showed that faster looming stimuli elicited a response in PPS neurons at farther distances as compared to slower stimuli. Some unpublished data from our lab (Noel, Galli, Blanke, Serino-Sound Velocity Modulates Audio-Tactile Peripersonal Space Boundary-in preparation) actually show an extended PPS boundary when audio-tactile interaction was probed with faster (75 cm/s) as compared to slower (25 cm/sec) sounds in humans, while utilizing the same paradigm as the one employed here. Finally, different participants partook in the two experiments, and perhaps subjects in Experiment 2 simply had on average a larger PPS representation (individual differences in PPS representation have been previously reported^17^,^18^).

#### Experiment 3. Peri-Hand PPS

Previous studies used the same audio-tactile paradigm to study PPS representation around the hand^13^. Here we applied a similar setup as in Experiment 1 to a new group of participants to measure the size of the peri-hand PPS. Participants responded to a tactile stimulus on the hand, while looming or receding sounds were presented as moving toward or away from the participant’s hand. Five distances were mapped (D1 through D5; 5, 27, 50, 71, and 93 cm) along a range of 100 cm. Participant’s hand was placed at shoulder-width and thus lateralized (not in the midline).

**Figure 3:**
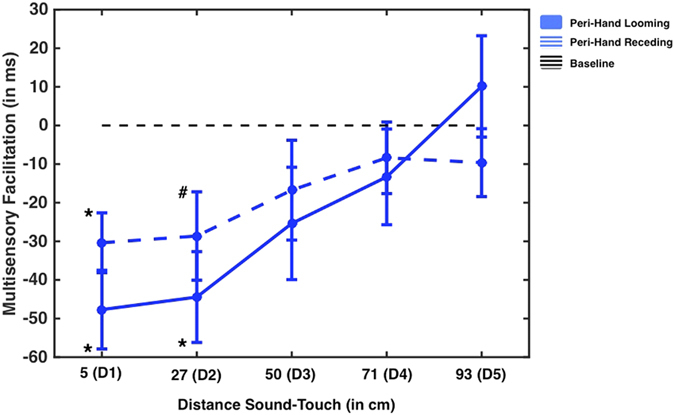
Peri-Hand Space-Experiment 3. Multisensory facilitation (in ms; negative values indicate multimodal audio-tactile RT <unimodal baseline tactile trials) as a function of sound distance at the moment of tactile stimulation (D1 indicates the smallest distance, while D5 indicates the largest distance) and the sound’s direction (RT to looming sounds are portrayed in solid blue and those due to receding sounds in dashed blue). The black dashed horizontal line indicates baseline, i.e., RT to the fastest unimodal tactile stimulation, *indicate a significant facilitation effect with respect to baseline (p < 0.05 Bonferroni-corrected, and # indicates a significant facilitation effect (p < 0.05) that did not survive multiple comparisons correction; . Error bars indicate +/−1 S.E.M.).

Baseline-corrected RTs were submitted to a 2 (Sound Direction: Looming vs. Receding) × 5 (Sound Distance: D1 through D5) Within-Subjects ANOVA. As depicted in Fig. 3, results revealed a significant Sound Direction × Sound Distance interaction (F(4, 60) = 7.064, p < 0.001, partial η^2^ = 0.320). In order to identify the source of the interaction, separate One-Way ANOVAs were conducted for each Sound Direction. The analysis performed solely on the looming stimuli, revealed a highly significant main effect of Sound Distance (F(4, 60) = 17.640, p < 0.001, partial η^2^ = 0.540). Bonferroni-corrected One-Sample t-test to baseline showed that audio-tactile RTs at D1 (5 cm) and D2 (27 cm) (but not at any of the farther distances; all correct p-values > 0.24) were statistically faster than RTs to the unimodal tactile condition (both p-values < 0.05, corrected). For the case of the receding sounds, the One-Way ANOVA also demonstrated a significant Sound Distance main effect (F(4, 60) = 3.138, p = 0.021, partial η^2^ = 0.173), which, however, appeared not as strongly as for the looming sounds (see slope of the two functions in Fig. 3 and compare the effect sizes for Sound Distance in looming and receding sound conditions). Bonferroni-corrected One-Sample t-test to baseline showed that during receding audio-tactile stimulation, only RTs at D1 (5 cm) were statistically faster than RTs to the unimodal tactile condition (p < 0.05 corrected). RT at D2 (27 cm) for receding sounds was also statistically faster than RTs at unimodal baseline, though this comparison did not survive correction for multiple comparisons (p = 0.024, uncorrected) (all other p > 0.35).

Thus, results from Experiment 3 show a significant modulation of tactile stimuli at the hand, when task irrelevant sounds are presented at D1 (5 cm) and D2 (27 cm), and not farther away. Not only looming, but also receding, sounds were found to affect tactile processing in a space-dependent manner, although such effect was stronger for looming than for receding stimuli^13^. We note that this differed from the results we obtained in Experiments 1 and 2, where only looming sounds affected tactile reaction times. Taken together, results from Experiment 1, 2 and 3 suggest that the size of PPS around the trunk is larger than that around the hand (i.e. the facilitation effect on tactile RT occurred between D3 and D4, that is about 43–62 cm, in Experiment 1 and between D2 and D3 (27–50 cm) in Experiment 3, which used the same setup – see Methods). In addition, the peri-trunk PPS representation, differently from the peri-hand PPS, is sensitive only to looming and not to receding stimuli. Thus, peri-trunk and peri-hand space seem to differ with respect to their extension into space and their response properties. We designed Experiments 4 and 5 to directly compare peri-hand and peri-trunk PPS.

#### Experiment 4. Peri-Trunk and Peri-Hand PPS

**Figure 4:**
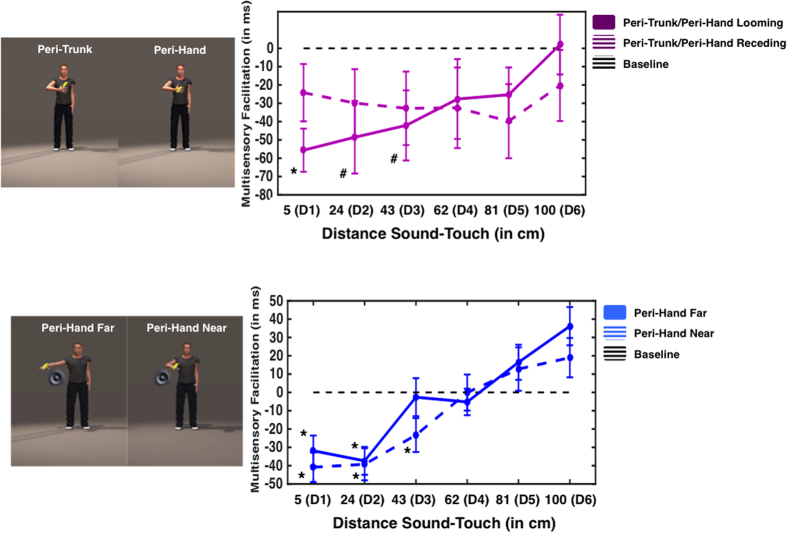
Peri-Hand and Peri-Trunk Space comparison. Upper panel – Experiment 4. Left upper panel: Participants received a tactile stimulation either on their chest (to study the peri-trunk space) or on their right hand (to study the peri-hand space), while the right hand was placed on the chest. Right upper panel: Multisensory facilitation (in ms; negative values indicate multimodal audio-tactile RT <unimodal baseline tactile trials) as a function of sound distance at the moment of tactile stimulation (D1 indicates the smallest distance, while D6 indicates the largest distance) and the sound’s direction (in solid for looming sounds and in dashed for receding sounds). RTs are averaged for chest and hand stimulation. The black dashed horizontal line indicates baseline, i.e., RT to the fastest unimodal tactile stimulation, * indicate a significant facilitation effect with respect to baseline (p < 0.05 Bonferonni-corrected) and # indicates a significant (p < 0.05) multisensory facilitation that did not survive correction for multiple comparisons; Error bars indicate +/−1 S.E.M. Lower panel – Experiment 5. Left lower panel: Participants received a tactile stimulation either on their right hand (to study the peri-hand space), while the right hand was placed either near the trunk or far from the trunk. Lower right panel; Multisensory facilitation (in ms; negative values indicate multimodal audio-tactile RT <unimodal baseline tactile trials) as a function of sound distance at the moment of tactile stimulation (D1 indicates the smallest distance, while D6 indicates the largest distance) and the posture of the hand with respect to the trunk (RT facilitation is represented in dashed when the hand was placed near the trunk, and in solid when the hand was placed far from the trunk). The black dashed horizontal line indicates baseline, i.e., RT to the fastest unimodal tactile stimulation, and * indicate a significant facilitation effect with respect to baseline (p < 0.05, Bonferroni-corrected). Error bars indicate +/−1 S.E.M. (The human figure was created by JNP, using ‘Poser 10′ software (http://my.smithmicro.com/poser-3d-animation-software.html)).

In order to directly compare the size of the peri-hand and the peri-trunk PPS, participants were asked to respond to tactile stimuli administered either to their right hand or to their trunk, while looming or receding sounds were presented. As illustrated in Fig. 4A (left panel), participants placed their right hand adjacent to, but not touching, their chest (at sternum level). In this way, in separate blocks, we assessed the PPS representation around the hand and around the trunk, while administering the same auditory stimulation. We reasoned as follows: if the hand PPS is fully independent from the trunk PPS, the speeding effect induced by the moving sounds should emerge at a closer distance for hand tactile stimulation (i.e., as in Experiment 3) than for trunk stimulation (i.e., as in Experiments 1 and 2). Conversely, if the hand and the trunk representations interact with each other, in the present experiment we expect a different pattern of audio-tactile interaction for hand stimulation when the hand is placed near the trunk, as compared to Experiment 3, when the hand was farther apart from the trunk.

Six distances were mapped over a range of 100 cm (5, 24, 43, 62, 81, and 100 cm). Baseline-corrected RTs were submitted to a 2 (Sound Direction: Looming vs. Receding) × 2 (Body Part: Hand vs. Trunk) × 6 (Sound Distance: D1 through D6) Within-Subjects ANOVA. Results revealed a significant interaction Sound Direction × Sound Distance (F(5, 85) = 10.065, p < 0.001, partial η^2^ = 0.372), whereas there was no interaction between Body Part, Sound direction and Distance (all p > 0.20), suggesting that the modulation of tactile processing due to the spatial position of sounds was comparable for tactile trunk and hand stimulation. In order to understand the nature of the Sound Distance × Sound Direction interaction, data were averaged across Body Parts, and then submitted to two separate ANOVAs, respectively for looming and receding sounds (see Fig. 4A). Looming sounds provoked a decrease in tactile reaction time as a function of proximity to the participant’s body (F(5, 85) = 4.303, p < 0.01, partial η^2^ = 0.202). Bonferroni-corrected multiple comparison analysis demonstrated that RTs at D1 (5 cm, p < 0.01, two-tailed corrected), at D2 (24 cm, p = 0.02, one-tailed) and at D3 (43 cm, p = 0.03, one-tailed) proved to be significantly different from baseline. Note that here one-tailed test can be used in this case, based on a-priori form Experiments 1–3. In the case of the receding sounds (similarly to results from Experiments 1 and 2) there was no Sound Distance effect (F (5, 85) = 2.018, p = 0.15).

Thus, when the hand was in close proximity to the trunk, that is within the spatial boundaries of the peri-trunk PPS, there was no evidence for a difference in the extension of PPS boundaries for hand and trunk stimulation (no interaction) and the extension of this combined peri-hand/trunk PPS was arguably most comparable to the size of the peri-trunk PPS measured in Experiments 1 (when performing a one-tailed test – however note that the correspondence between findings in Experiment 1 and 4 are not exact when performing 2-tailed comparisons). In addition, such peri-hand/trunk PPS was insensitive to receding sounds, as it was the case for the peri-trunk PPS in Experiment 1, and differently from the case of the peri-hand PPS in Experiment 3. Thus, it could be conceived that when the hand was placed on the midline and near the trunk, the peri-hand representation was encapsulated by the peri-trunk representation – this is however not so say that the selective representation of the peri-hand space is absent, but rather that it cannot be captured by the present behavioral experimental approach.

#### Experiment 5. Peri-hand PPS outside and inside the peri-trunk PPS

The size of the peri-trunk PPS (Experiments 1 and 2) was bigger than that of the peri-hand PPS (Experiment 3), whereas when they were directly contrasted (Experiment 4), there was no difference between the extents of the two PPS representations. We note, however, that the position of the hand with respect to the trunk differed between Experiment 3 (distant from the trunk – at shoulder-width) and 4 (on the midline). Based on these data, we hypothesized that when the hand is placed within the spatial boundaries of the trunk PPS, multisensory stimuli directed toward the hand and trunk may be coded as related to a common or integrated reference frame, centered on the trunk (as seemingly suggested by the dimensions of the PPS in Experiment 4). However, when the hand is placed farther from the trunk, we hypothesized that multisensory stimuli directed to the hand should be coded as referenced to the hand (compatible with results obtained in Experiment 3). In Experiment 5 we tested this prediction by directly comparing the size of the peri-hand PPS with the hand either placed close to the trunk (peri-hand near condition) or farther apart (peri-hand far condition; see Fig. 4B, left panel). Importantly, the location and distance of auditory stimuli was kept constant in the two conditions, by placing the sound source in between the two hand positions. Six different sound distances were sampled over a range of 100 cm (5, 24, 43, 62, 81, and 100 cm), with looming sounds only.

Baseline-corrected RTs from audiotactile trials were submitted to a 2 (Hand Posture: Near vs. Far) × 6 (Sound Distance: D1 through D6) Within-Subjects ANOVA. Results revealed a significant Hand Posture × Sound Distance Interaction F(5, 90) = 3.747, p < 0.01, partial η^2^ = 0.172), which is illustrated in Fig. 4B. Separate One-Way ANOVAs showed that both in the Peri-Hand Near and in the Peri-Hand Far conditions there was a significant effect of Sound Distance (F(5, 90) = 47.165, p < 0.001, partial η^2^ = 0.724 and F(5, 90) = 33.140, p < 0.001, partial η^2^ = 0.648, for Peri-Hand Near and Peri-Hand Far, respectively). However, the modulation of tactile RTs as a function of sound distance was different for the two hand postures. Multiple comparisons corrected One-Sample t-tests revealed that in the Peri-Hand Near condition D1, D2, and D3 (5, 24 and 43 cm) all differed from baseline (p < 0.05, corrected), whereas only D1 and D2 (5 and 24 cm) did in the Peri-Hand Far condition. Thus, the boundary of PPS around the hand was more extended in space when the hand was placed close to the trunk (i.e. between D3 and D4 – 43 and 62 cm, as in Experiment 1) as compared to when it was placed far apart (i.e. between D2 and D3 – 24 and 43 cm, as in Experiment 3), confirming the findings from Experiments 3 and 4, but now in the same subjects sample.

Taken together, results from experiments 1–5 show that two different PPS representations exist, one for the peri-hand and another one for peri-trunk space and that they vary in their extension into space and for their selectivity to stimuli direction (looming vs. receding sounds). However, these two representations do not appear to be independent from each other. Rather, it seems that the size and the direction tuning of the peri-hand varies depending on the distance of the hand from the trunk, suggesting that the peri-hand space is coded with respect to the peri-trunk space.

### Section 2. Relationship between peri-trunk and peri-face PPS

#### Experiment 6. Peri-face and Peri-trunk PPS

**Figure 5:**
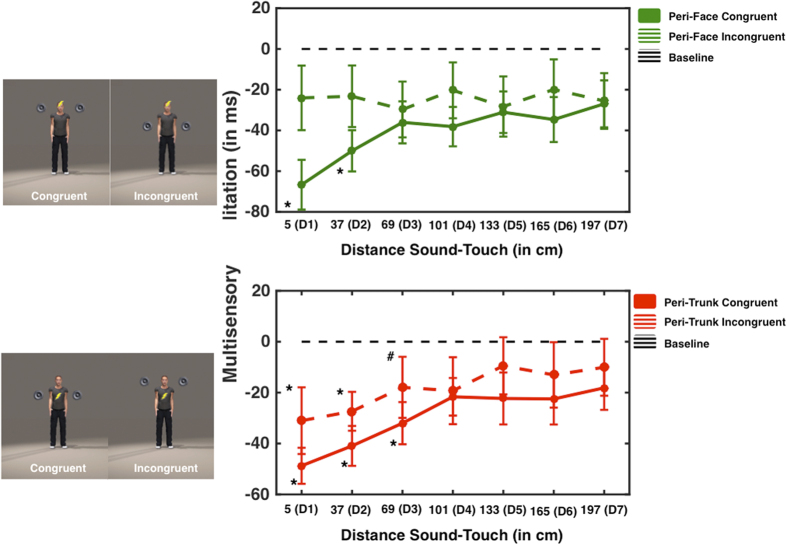
Peri-Face and Peri-Trunk comparison-Experiment 6. Left upper panel: Participants received a tactile stimulation on their forehead (to study the peri-face space), while sounds (depicted by the couple of loudspeakers) approached their face (congruent audio-tactile stimulation) or their trunk (incongruent audio-tactile stimulation). Right upper panel: Multisensory facilitation (in ms; negative values indicate multimodal audio-tactile RT <unimodal baseline tactile trials) as a function of sound distance at the moment of tactile stimulation (D1 indicates the smallest distance, while D6 indicates the largest distance) and congruency condition of audio-tactile stimulation (solid line represents the congruent condition, and dashed line the incongruent condition). The black dashed horizontal line indicates baseline, i.e., RT to the fastest unimodal tactile stimulation, and * indicate a significant facilitation effect with respect to baseline (p < 0.05, Bonferroni-corrected). Error bars indicate +/−1 S.E.M. Left lower panel: Participants received a tactile stimulation on their chest (to study the peri-trunk space), while sounds approached their trunk (congruent audio-tactile stimulation) or their face (incongruent audio-tactile stimulation). Right lower panel: Multisensory facilitation (in ms; negative values indicate multimodal audio-tactile RT <unimodal baseline tactile trials) as a function of sound distance at the moment of tactile stimulation (D1 indicates the smallest distance, while D6 indicates the largest distance) and congruency condition of audio-tactile stimulation (solid line represents the congruent condition, and dashed line the incongruent condition). The black dashed horizontal line indicates baseline (RT to tactile stimulation), *represent a significant difference between multimodal and unimodal conditions at a particular sound-touch distance (p < 0.05, Bonferroni-corrected), # represents a significant multisensory facilitation (p < 0.05) that did not survive correction for multiple comparison, and error bars indicate +/−1 S.E.M. (The human figure was created by JNP, using ‘Poser 10′ software (http://my.smithmicro.com/poser-3d-animation-software.html)).

In Experiment 6 we tested whether differences exist between the peri-trunk and another classical PPS representation, the peri-face space. As it is not possible to physically displace the face from the trunk (as done between hand and trunk) we manipulated the association between the two representations by administering the tactile target stimulus either to the trunk or the face, while sounds moved either toward (or away from) the trunk or toward (or away from) the face, in a 2 × 2 factorial design. In this way, we mapped the peri-face and the peri-trunk space representation, while dynamic auditory stimuli approached either the mapped body part (congruent audio-tactile stimulation) or another body part (incongruent audio-tactile stimulation) (see Fig. 5). We reasoned that if the face and the trunk PPS representations are segregated and independent from each other, only congruent audio-tactile stimulation should induce a spatially dependent modulation of tactile processing for the stimulated body part. Rather, if the two representations interact or are integrated, also incongruent audio-tactile stimulation might induce significant spatially dependent modulation of tactile responses. Seven distances were mapped with both looming and receding sounds, over a range of 200 cm (5, 37, 69, 101, 133, 165, and 197 cm).

Baseline-corrected RTs were submitted to a 2 (Sound Direction: Looming vs. Receding) × 2 (Sound Location: Face vs. Trunk) × 2 (Touch Location: Face vs. Trunk) × 7 (Sound Distances) mixed ANOVA (Sound Direction being a within-subjects variable, while Sound Location and Touch Location being between-subject variables). Results revealed a significant main Sound Direction × Sound Distance × Sound Location × Touch Location (F(6, 342) = 4.510, p < 0.001, partial η^2^ = 0.076) interaction. In order to understand the source of this interaction, two separate 2 (Sound Location) × 2 (Touch Location) × 7 (Sound Distance) mixed ANOVAs were conducted for each Sound Direction. In the case of Looming sounds, the ANOVA revealed a significant Sound Location × Touch Location × Sound Distance interaction (F(6,330) = 4.775, p < 0.001, partial η^2^ = 0.80). Therefore, in order to explore this interaction, two separate 2 (Sound Location) × 7 (Sound Distance) ANOVAs were conducted for each Touch Location (Face or Trunk). When touch was given on the face, we found a Sound Location × Sound Distance interaction (F(6, 180) = 4.882, p < 0.001, partial η^2^ = 0.140), as depicted in Fig. 5A. Separate One-Way ANOVAs for each Sound Location revealed that, when both Touch and Sound were given to the face, the main effect of Sound Distance was significant (F(6, 90) = 18.274, p < 0.001, partial η^2^ = 0.549). Bonferroni-corrected multiple comparisons to baseline showed that RTs at D1 and D2 (5 and 37 cm, all p < 0.05), but not at farther distances (all p-values > 0.26), were significantly faster than under the unimodal tactile condition. On the other hand, when sound approached the trunk, while touch was given to the face, the One-Way ANOVA showed no Sound Distance main effect (F(6, 90) = 0.258, p = 0.955, 1−β = 0.116).

Results for the case when touch was given on the trunk are shown in Fig. 5B. The 2 (Sound Location) × 7 (Sound Distance) mixed ANOVA revealed a significant main effect Sound Distance (F(6, 150) = 8.204, p < 0.001, partial η^2^ = 0.247), yet did not show evidence for a Sound Distance × Sound Location interaction (F(6, 150) = 1.450, p = 0.199, 1–β = 0.551), differently from what found for the same comparison when the touch was given on the face (see Fig. 5A). This pattern of results implies that when a touch was applied to the trunk, sounds approaching either the trunk or the face evoked the same spatially dependent modulation of auditory stimuli on tactile processing. In order to confirm this finding (and not conclude on the basis of a null effect), individual One-Way ANOVAs were performed for each Sound Location level. As depicted in Fig. 5B, when both touch and sound were congruently directed to the trunk, there was a significant Sound Distance main effect (F(6, 84) = 8.077, p < 0.001, partial η^2^ = 0.366). Bonferroni-corrected multiple comparisons to baseline revealed that RTs were faster than unimodal tactile RTs when the sound was at D1, D2, and D3 (5, 37, and 69 cm, all p < 0.05, corrected). Similarly, when sounds were directed at the face, with tactile stimuli applied to the trunk, the One-Way ANOVA revealed a significant Sound Distance main effect (F(6, 66) = 2.335, p = 0.042, partial η^2^ = 0.175). Bonferroni-corrected multiple comparisons to baseline showed that RTs when the sound was at D1 and D2 (5 and 37 cm) were significantly different from unimodal baseline (all p < 0.05, corrected). The comparison at D3 (69 cm) was also significantly different from 0, yet did not survive correction (p = 0.026, uncorrected). The analyses performed on the receding sounds showed no main effects, nor any interaction (all p > 0.15; as in Experiments 1, 2, and 4).

To summarize, in the case of tactile stimulation of the trunk, auditory stimuli congruently directed toward the trunk or incongruently directed toward the face, equally speeded up, in a spatially dependent manner, tactile RT. In both cases, the critical distance at which approaching sounds elicited a significant speeding effect on tactile processing was between D3 and D4 (69 and 101 cm). We note that this distance is comparable to the boundaries of the peri-trunk PPS assessed in Experiments 1, 2, and 4 (43–62 cm in Experiment 1 and 4, 80–95 cm in Experiment 2). In contrast, the perception of tactile stimuli on the face was affected only by congruent auditory stimuli, i.e. only by sounds directed toward the face and by not those directed toward the trunk. In the case of congruent audio-tactile stimulation on the face, the speeding effect started when sounds were between D2 and D3 (37 and 69 cm), suggesting that the boundary of the face PPS is closer to the body than that of trunk PPS.

#### Experiment 7. Peri-trunk and Peri-face PPS shown by visuo-tactile interaction

In all previous experiments, we tested audio-tactile interactions to investigate the PPS representation. However, many previous studies of PPS focused on visuo-tactile rather than on audio-tactile interaction. In order to independently confirm and verify that the PPS representation as investigated here is not limited to audio-tactile interaction and also extends to visuo-tactile interaction, we next studied the effects of a moving visual stimulus on tactile processing. Tactile targets were administered either to the face or to the trunk (as in Experiment 6), while looming visual stimuli, directed toward the face, were presented in a Virtual Reality (VR) environment through a head-mounted display (HMD). Seven distances were sampled (5, 37, 69, 101, 133, 165, and 197 cm). The effect of congruency could not be tested as the size of the visual field did not allow for presentation of incongruent stimuli (such as face touches associated with looming visual stimuli approaching the trunk).

**Figure 6:**
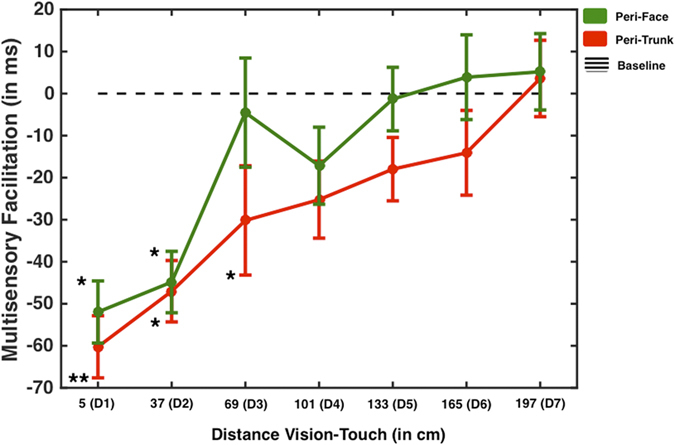
Visuo-Tactile Peri-Face and Peri-Trunk representation – Experiment 7. Mean RT difference between multisensory (visuo-tactile) and unisensory (tactile) trials as a function of the distance where a virtual ball was presented at the time of tacile stimulation. Green solid line shows the RT profile when touch was administer to the face (to study the peri-face space), while the red solid line shows the RT profile for when tactile stimulation was given on the chest (to study the peri-trunk space). The black dashed horizontal line indicates baseline (RT to tactile stimulation), * and ** represent a significant difference between multimodal and unimodal conditions at a particular sound-touch distance (respectively, p < 0.05 and p < 0.01, Bonferroni-corrected), and error bars indicate +/−1 S.E.M.

A 2 (Touch Location: Face vs. Trunk) × 7 (Visual Distance) Within-Subjects ANOVA was carried out on baseline-corrected RTs. Results revealed a significant Touch Location × Visual Distance interaction (F(6, 114) = 2.405, p = 0.032, partial η^2^ = 0.112). Separate One-Way ANOVAs for each Touch Location confirmed that in both cases, when touch was given on the face or on the trunk a Visual Distance main effect was observed (Face: F(6, 114) = 21.834, p < 0.001, partial η^2^ = 0.535; Trunk: F(6, 114) = 25.310, p < 0.001, partial η^2^ = 0.571). As Illustrated in Fig. 6, the Touch Location × Visual Distance interaction is explained by the fact that when touch was given on the face, RT at only D1 and D2 (5 and 37 cm) were statistically different from baseline (p < 0.05, corrected), while when touch was given to the trunk, RT at D1, D2, and D3 (5, 37 and 69 cm, p < 0.05, corrected) were faster than baseline (One-Sample t-tests, Bonferroni corrected for multiple comparisons).

Thus, the results of this last experiment showed that a spatially dependent multisensory effect on tactile processing can be identified by presenting visual stimuli, and not only auditory stimuli, as in the previous six experiments. In addition, results from Experiment 7 also replicate, with visuo-tactile stimulation, the main findings from Experiment 6 comparing peri-trunk and peri-face representations with audio-tactile stimulation. Indeed, the PPS boundaries were again identified at a greater distance from the body for trunk stimulation, as compared to face stimulation.

### Section 3. Comparison of the size of PPS boundaries for different body parts

Results from the previous experiments show that the size of PPS representation varies around different body parts. In order to estimate the location of the PPS boundaries around the different body parts on a metric scale, we transformed the D values from the different experiments (1–6) in cm, and then we fitted all data corresponding to a particular body part across experiments, to a linear and sigmoidal curve (for details see Canzoneri *et al.*, 2012^13^).

**Figure 7:**
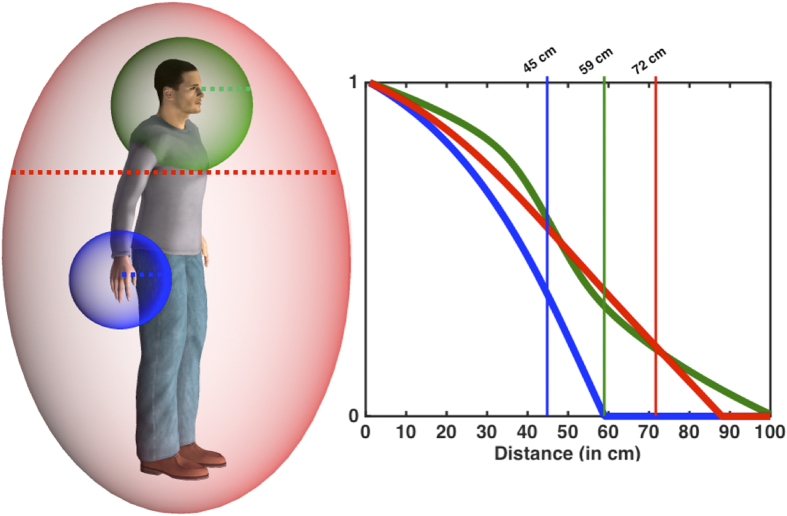
Left panel: Schematic representation of the relative size of peri-hand (blue), peri-face (green), and peri-trunk (red) representation (Notice the gradient in the depiction of peri-hand and peri-face representation: data was not collected for the back space for the hand and the face and hence this panel is to be taken simply as a conceptual schema. Dashed lines represent spatial extensions that were actually probed in this study). Right panel: Estimates were obtained by fitting all data corresponding to a particular body part across studies 1–6 to a sigmoidal curve after adjusting for total space mapped and points sampled. In this manner, regardless of the total amount of space sampled (1 or 2 meters) or the total amount of distances sampled (5, 6, or 7), the central point parameter of the sigmoid function (peri-hand and peri-face) or the intersection between the linear function describing audio-tactile RTs and the lower bound of the 95% confidence interval describing tactile RTs (in the case of the peri-trunk) were extracted as a punctuate estimate of peri-hand (~45 cm), peri-face (~59 cm), and peri-trunk (~72 cm) space. (Figure drawn by JPN; the human was created with Poser 9, SmithMicro Software, lincense: XF90CRD-0001-14YT-QJF0-201X-EBL7).

In terms of the peri-hand representation, as previously noted the sigmoidal fitting explained significantly more variance (R^2^ = 0.69 +/−0.03) than the linear (R^2^ = 0.50 +/−0.04; p = 0.039) did. This was also the case for the peri-face representation (sigmoidal; R^2^ = 0.66 +/−0.02, linear; R^2^ = 0.53 +/−0.03; p = 0.044), but not for the peri-trunk representation (sigmoidal; R^2^ = 0.60 +/−0.05, linear; R^2^ = 0.65 +/−0.03; p = 0.18). As the sigmoidal fitting was best for hand and face, and served equally well as the linear fitting for the trunk, the central point of the sigmoidal fitting was estimated as a proxy of the location where tactile RT were significantly boosted by sound presentation for the hand, face, and trunk. As illustrated in Fig. 7, the fitting procedure demonstrated that PPS boundaries were smallest for the peri-hand (i.e. 45 cm +/−7 cm; Experiments 3, 4, 5) intermediate for the peri-face space (i.e., 59 cm +/−6 cm; Experiment 6 and 7) and largest (i.e., 72 cm +/−7 cm) for the peri-trunk space (Experiments 1, 2, 4 and 6) (see Figure caption for details).

## Discussion

We performed the first extensive mapping in humans of the representation of PPS around three different body parts; hand, face, and trunk. We estimated the critical distance in space where task irrelevant auditory or visual cues significantly boosted processing of tactile information on the body. This distance can be considered the boundary of PPS representation in humans, providing a proxy of the extent of hypothetical receptive fields of multisensory integration systems mapping the space around the body.

We obtained two main findings. First, the PPS boundary was estimated at different distances from the body, being smallest for the hand, larger for the face and largest for the trunk (Fig. 6). In addition, in case of hand stimulation, both approaching and receding stimuli showed space-dependent modulations of tactile processing, whereas only approaching stimuli did so for face and trunk stimulation. These results suggest that at least three body-part specific PPS representations exist, differing for extension and directional tuning. Second, PPS representations for the hand, face, and trunk are not fully independent from each other, but interact in such a way suggesting that hand and face PPS are referenced to the more global trunk-centered PPS representation.

Our findings, indicating different extensions and direction selectivity of PPS around different body parts in humans, are compatible with neurophysiological data in monkeys, where PPS neurons are characterized by a close relationship between the sizes of their tactile and visual or auditory RFs. Most neurons in area 7 have extensive tactile RFs, even covering the whole body, often bilaterally. Their visual RFs also extend bilaterally over large regions of the visual field, up to more than 1 m^19^,^20^,^21^,^22^. Neurons in area VIP show tactile RFs usually centered on the head or on the upper trunk, and their visual^4^ or auditory^23^ RFs are usually limited to the upper space (range: 10–60 cm). Most neurons in the premotor cortex, with tactile RFs on the arm, have smaller visual RFs, normally extending for 5 or 20 cm^1^ and auditory RFs of ~30 cm^24^. PPS neurons also show a directional tuning, being in general more sensitive to looming than to receding stimuli^2^,^3^. We extend these data by reporting that the degree of preference for looming stimuli varies for different body parts. For the hand, both looming and receding sounds affected tactile detection, although receding sounds induced a less defined spatial gradient (Exp. 3; Canzoneri *et al.*,2012^13^). Instead, for face and trunk stimulation, only looming stimuli induced a space-dependent modulation of tactile RT (Experiments 1, 2, 6 and 7).

Our proposal that there exists separate, differently sized and directionally tuned, body-part specific PPS representations in humans is compatible with the most commonly accepted function of PPS, i.e., a multisensory-motor interface for body-object interaction. Whether processing visual or auditory signals in order to plan an approaching movement^25^, or in order to react to potential threats^1^, these neural representations have been argued to code selectively for those areas of space that are most relevant for interactions^26^,^27^. Different body parts interact with objects over different portions of space: hands-object interactions occur within a limited space around the arm^28^, face-objects interactions mainly occur in the context of bringing-to-mouth actions within the upper space^29^,^30^ whereas trunk-objects interactions materialize in a larger portion of space and are related to whole-body actions, e.g., walking^15^. Thus, multisensory RFs of PPS neurons may develop around tactile RF with a size appropriate to anticipate potential interactions between an external stimulus and a specific body part. In addition, due to the large repertoire of upper-limb actions, the hand usually receives touches both from colliding stimuli and receding stimuli. Conversely, it is much more likely that face or trunk tactile stimulation originates from an approaching stimulus. These differences in body parts-object interactions might explain the differences, not only in the size, but also in the directional tuning of PPS representations around the hand, face and trunk found in the present study.

The second main finding of our study is that separate PPS representations anchored to hand, face, and trunk are not fully independent from each other. Several data support this conclusion. First, the extent of the hand PPS varied depending on the spatial relationship between the hand and trunk. The hand PPS was smallest when the hand was placed laterally and farther away from the trunk (Exp. 3, 5), enlarged when the hand was placed close to the trunk (Exp. 5) and became indistinguishable from the peri-trunk space (Exp. 4), when the hand was placed on the trunk. The directional sensitivity of the peri-hand PPS also depended on the hand-trunk distance: when the hand was placed far apart from the trunk, both looming and receding sounds modulated hand tactile responses (Exp. 3), whereas when the hand was on the trunk, no effect of receding stimuli was found (Exp. 4), meaning that the hand PPS showed the same directional tuning as the trunk PPS (Exp.1, 2, 6 and 7). These findings suggest that although a specific hand PPS exists, it can be integrated with the larger and differently direction-tuned trunk PPS, so that the former is indistinguishable from the latter when the hand is placed on the trunk. A similar conclusion is supported by the comparison between the peri-face and the peri-trunk representations. Results from Experiments 6-7 show that sounds directed either toward the face or the trunk equally modulated tactile responses for the trunk, whereas, only sounds traveling congruently toward the face affected facial tactile responses. Thus, stimuli approaching the face or the trunk are referenced to a more extended PPS representation around the trunk, whereas stimuli directed to the trunk are not integrated in the smaller peri-face PPS, suggesting the face PPS is also integrated within the trunk PPS.

In sum, hand- and face-centered PPSs are referenced to the trunk-centered PPS, which we found to be a more extended representation of the space surrounding the body. These findings are in line with the computational mechanisms underlying reference frame transformation constructing multisensory receptive fields around specific body parts^31^,^32^,^33^. In order to construct a visual RF anchored to the head, it is necessary to take into account the position of the eye and the head direction, while the arm position is irrelevant. Accordingly, the responses of VIP neurons, mapping the peri-head space, vary depending on the position of the eyes and the head, but not of the arm^34^. For an arm-anchored visual RF, it is necessary to compute the position of the arm relative to the eye, head and trunk^34^. The response of hand PPS neurons is modulated by eye, head and arm position^2^,^3^. In contrast, head position and arm position are irrelevant for a trunk-centered spatial representation. Accordingly, responses of area 7b neurons (mapping the trunk or the whole body) are not modulated by arm and head movements^22^,^35^. Thus, the trunk PPS constitutes a larger, more general representation of the space around the body which other smaller body-part centered representations are referenced to.

We speculate that the trunk-centered PPS constitutes a whole-body reference frame relative to which a global, egocentric representation of space is formed. Several arguments support this view. First, most body parts are anatomically connected to the trunk, which is “the great continent of the body, relative to which all other body parts are mere peninsulas”^36^. Such anatomical constraints determine the way different reference frames are computed relatively to each other^31^,^32^. Neuropsychological studies on right brain damaged patients with visuo-spatial neglect suggest that a trunk-centered representation of space is fundamental for coding a global, egocentric reference frame that defines the location of external stimuli with respect to the subject. Neglect patients typically present a bias in under-representing the left side of space and over-representing the right side. Such left-right asymmetry is defined with respect to the trunk midline^37^,^38^,^39^, suggesting that the trunk grounds the subjective center of spatial perception. Extending this notion, here we propose that the trunk PPS, by integrating not only body-related signals, but also information related to external stimuli potentially interacting with the body, in a global, egocentric reference frame, may also constitute a basic neural representation that is of relevance for the self and self-consciousness^40^,^41^,^42^,^43^,^46^.

## Methods

### Participants

A total of 191 subjects (65 females, mean age = 24.2, range: 18–33) partook in a total of 7 experiments, in addition to 3 methodological control experiments (see supplementary information). Fifteen subjects (6 females, mean age = 25) participated in Experiment 1, sixteen (6 females, mean age = 24) in Experiment 2, sixteen (5 females, mean age = 21) in Experiment 3, eighteen (7 females, mean age = 24) in Experiment 4, nineteen (7 females, mean age = 24) in Experiment 5, sixty in Experiment 6 (18 females, mean age = 25), and twenty (6 females, mean age = 24) in Experiment 7. Note that for all the of experiments we used a within-subjects design, whereas for Experiment 6 we adopted a between-subjects design, and this explains the difference in sample size used in Experiment 6. Twenty-seven (10 females, mean age = 23) participants took part in 3 methodological control experiments.

All participants reported normal touch and hearing and had normal or corrected-to-normal visual acuity. All participants gave their informed consent to take part in this study, which was approved by the local ethics committee – The Brain Mind Institute Ethics Committee for Human Behavioral Research at EPFL – and conducted in line with the Declaration of Helsinki. All participants were remunerated with 20 Swiss Francs for their time.

### Stimuli and Apparatus

#### Tactile Stimuli (Experiments 1–7)

A vibrotactile device consisting of a small vibrating motor (Precision MicroDrives shaftless vibration motors, model 312–101, 3 V, 60 mA, 9000 rpm, 150 Hz, 5 g) was utilized in order to administer tactile stimuli. The motor had a surface area of 113 mm^2^ and reached maximal rotation speed in 50 ms. The placement and the number of vibrators used varied according to experimental needs. A single vibrotactile device was placed on the upper chest (sternum level) of participants for Experiment 1, and Experiments 4, 6, and 7 (peri-trunk condition for the latter three). Two vibrotactile devices were placed on the trunk of participants (sternum level, front and back) for Experiment 2. Tactile stimulation was delivered to the hand (dorsum), by means of a single vibrotactile device, in Experiment 3, 4 (peri-hand condition), and 5. Finally, a vibrotactile device was placed on the forehead of participants in Experiments 6 and 7 (peri-face condition). Tactile stimulation always lasted 100 ms.

#### Acoustic Stimuli (Experiments 1–6)

Two different setups were used in order to administer auditory stimulation; a 2-speakers setup and a 16-speakers setup depending on experimental needs (see Fig. 1 left panels in supplementary information). The 2-speaker setup was utilized in Experiment 1, 3, 4, and 5. Six body-sound distances were sampled and sounds traveled at a velocity of 22 cm/s in Experiment 1, 4, and 5. Five distances were sampled, and sound traveled at a velocity of 22 cm/s in Experiment 3. Distance between the far and near speaker was always 100 cm. As in Canzoneri *et al.*, 2012^13^ the apparent sound position was given by the differential sound intensity between the two speakers, as well as the relative position between each other and to the participant. In this manner, even with only 2 speakers, a continuous dynamic looming or receding sound was generated, and thus the points in space “sampled” (audio-tactile distances) were dictated by the temporal offsets used between sound onset and tactile onset (e.g., in the case of a looming sound, the longer the auditory and tactile onset discrepancy, the closer the sound). Validation of this set-up is described extensively in Canzoneri *et al.*, 2012, and the same setting was effectively used in several successive studies^14^,^44^,^45^. In supplementary material we report new data supporting the effectiveness of the implemented manipulation in inducing a clear sensation of sounds moving in space and being perceived at a different distance from the participants’ hand or trunk, for the different temporal delays (see *Validation 2-speaker setup* section in Supplementary Information). The 16-speaker setup was utilized in Experiments 2 and 6. Fourteen body-sound distances were sampled in Experiment 2 (7 in the front, 7 in the back, while 7 distances were sampled in Experiment 6 (all in the front space). The 16-speaker setup produced dynamic sounds moving at 35 cm/s in Experiment 2, and 25 cm/s in Experiment 6. In Experiment 2 participants stood halfway down the array of speakers (as to have 1 meter of speaker toward the front and toward the back), while for Experiment 6 subjects stood at the end of the array and hence the farthest speaker was at 2 meters. Looming and receding sounds were utilized in Experiment 1, 2, 3, 4, and 6, while only looming sounds were employed in Experiment 5. See Supplementary Material online for details and validation of the acoustic stimuli.

#### Visual Stimuli (Experiment 7)

A tridimensional virtual ball looming toward participants’ face was used in Experiment 7. This ball travelled approximately 2 meters in virtual space at a velocity of 100 cm/s until making fictive contact with the participant’s face. The virtual ball was presented on a head-mounted display (HMD, VR1280 Virtual Research Systems, Inc., 1280 × 1024 pixels, 60-degree diagonal field of view, 60 Hz) and rendered by means of in-house software (ExpyVR; http://lnco.epfl.ch/expyvr).

### General Procedure

Upon arrival at the laboratory vibrotactile device(s) were placed on the participant. The number of vibrators and their location on the participant’s body varied according to experimental demands (see above). Subjects were informed that they would feel a tactile vibration and hear sounds approaching toward and/or receding from them (or they would see a virtual ball in Experiment 7). They were informed that sounds (or the visual stimuli, in Experiment 7) were task-irrelevant, and that their task was to respond as accurately and rapidly as possible to tactile vibration by pressing a particular button on a wireless gamepad (XBOX 360 controller, 125 Hz Sampling Frequency, Microsoft, Redmond, WA), They were also informed that in some trials (catch trials) only sounds without tactile stimulation would be presented, and yet on other trials (baseline trials) only tactile vibration would be administered (see below for detailed breakdown of trials).

### Design and Analysis

All experiments included three types of trials. Approximately 70% of trials were experimental bimodal trials in which participants heard a sound (or saw a moving ball in Experiment 7), either approaching toward or receding from them, and, at a given moment in time (hereafter T), they received a 100 ms vibrotactile stimulus. Participants were to respond to touch as rapidly as possible. When presented with experimental bimodal receding trials, the stimuli temporal dimension, i.e. the delay between sound onset and touch delivery, and the spatial dimension, i.e., the distance between the location of sound and the body when touch is given, map onto each other positively and linearly. That is, tactile stimulation onset at increasing temporal delays (T1, T2 etc…) corresponded to sounds being perceived at increasing distances (hereafter D) from the body, (D1, D2, etc…). Contrarily, when subjects were presented with looming sounds (which therefore by definition start far and over time come closer to the participant), the stimuli temporal and spatial dimensions map negatively and linearly. That is, D1 and D2 respectively correspond to the last and penultimate temporal delays, and so forth.

The aim of the task is to identify the farthest distance from the body (D) at which sounds or visual stimuli significantly affected tactile processing, that is when audio-tactile RTs (or visuo-tactile RTs in Experiment 7) become significantly faster than responses to tactile stimulation alone. Such distance is taken as a proxy of the boundary of PPS representation. Thus, we included also 20% of unimodal tactile trials in which a vibrotactile stimulus was delivered in the absence of auditory (or visual) stimulation. For sake of experiment duration, unimodal tactile trials were administered at two different temporal delays, corresponding to the equivalent time of the nearest and farthest distance sampled during experimental trials (see Canzoneri *et al.*, 2012^13^ for a similar approach). Unimodal tactile trials are considered baseline trials and were used to show a multisensory facilitation effect on tactile RT due to sounds presented within the PPS as compared to RT to unimodal tactile stimuli. To this aim, RT in the audio-tactile stimulation conditions were corrected, on an individual basis, for baseline performance. That is, for each participant, we identified the baseline condition resulting in the fastest RT among the baseline unimodal tactile conditions, we calculated the mean raw RT for that condition, and that value was subtracted from the mean raw RT to the tactile stimulus for each audio-tactile (or visuo-tactile) condition. In this way, we adopted the most conservative criterion to show a facilitation effect on tactile RT due to sound presentation and spatial location as compared to the fastest unimodal tactile RT. Negative deviations from the baseline (which by definition is zero) indicate a multisensory facilitation effect. In order to identify the boundaries of PPS representations, we searched for the farthest point in space where sound induced a significant facilitation effect as compared to baseline (i.e., the fastest unimodal tactile condition) (see also Noel *et al.*, 2015^15^ for a similar approach). Baseline trials were also useful for other purposes. Firstly, they were used to control for spurious modulation in reaction times due to an expectancy effect (i.e., the fact that if a trial has started a moment ago and no tactile vibration has been given, it is more and more likely that the tactile stimuli is approaching in time). In addition, computing deviations from a unimodal tactile condition allows for comparisons between subjects and across experiments, as it controls for between-subjects difference in RT to tactile stimuli. This approach also allows controlling for differences in tactile sensitivity between different body parts. Since we always identified PPS boundaries by comparing unimodal tactile responses with audio-tactile or visuo-tactile conditions within the same body part, we excluded that differences PPS extension would depend on differences in response to touch between the tested body parts.

Finally, approximately 10% of trials were unimodal auditory (or visual in Experiment 7) trials, and thus catch trials, due to the task request. In these trials only a sound (or a virtual ball) was presented (either looming toward or receding from the participant) but no tactile stimuli were given. Participants were to withhold from responding. Catch trials were included in order to avoid entrainment of an automatic motoric response and to assure that participants were attentive to the task.

For every experiment, preliminary analyses were conducted on unimodal auditory/visual catch trials to test for accuracy in the tactile task. Due to the settings of tactile target stimulation, participants were always very accurate in the task, and there were no statistical differences in response accuracy between the different conditions in any experiment. Thus, performance is always analyzed in terms of RT only^13^,^14^,^15^,^44^,^45^,^46^. Accuracy data (i.e. percentage of correct withholding of response during catch trials and 1-percent omission during experimental trials) for the different experiments are reported in Table 1.

The total number and the combination of trials varied according to number of distances sampled, total amount of space sampled, and sound velocity. Experiment 1 consisted of 240 trials ([multisensory (D1 through D6) + baselines (D1 and D6) + catch trials] × 2 (looming and receding) × 16 repetitions). Experiment 2 consisted of 372 trials ([multisensory (13 distances) × 2 sound directions + 3 baselines + 2 catch trials] × 12 repetitions). Experiment 3 consisted of 208 trials ([multisensory (D1 through D5) × 2 (looming and receding) + baselines (D1 and D5) + catch trials] × 16 repetitions). Experiment 4 consisted of 480 trials ([multisensory (D1 through D6) × 2 (looming and receding) + baselines (D1 and D6) + catch trials] × 2 (peri-hand and peri-trunk) × 16 repetitions). Experiment 5 consisted of 432 trials ([multisensory (D1 through D6) + baselines (D1 and D6) + catch trials] × (Near vs. Far) × 24 repetitions). Experiment 6 consisted of 320 trials ([multisensory (D1 through D7) + baselines (D1 and D7) + catch trials] × (looming and receding) × 16 repetitions). Finally, Experiment 7 consisted of 320 trials ([multisensory (D1 through D7) + baselines (D1 and D7) + catch trials] × 2 (Trunk vs. Face) × 16 repetitions). Inter-trial intervals were randomly shuffled between 500 and 700 ms. The experiments never exceeded 60 minutes.

### Fitting Procedure

In order to provide a concise summary across experiments (which differed in points in space sampled, total distance sampled, and speed of incoming and outgoing stimuli), data from all 7 experiments were fitted to both sigmoidal and linear curves. In order to compare across the two fitting procedures, the sigmoidal curve was limited to two parameters as in Canzoneri *et al.*, 2012^13^. The results from this fitting procedure are shown in Fig. 7 as a conceptual schematic.

## Additional Information

**How to cite this article**: Serino, A. *et al.* Body part-centered and full body-centered peripersonal space representations. *Sci. Rep.* **5**, 18603; doi: 10.1038/srep18603 (2015).

## Supplementary Material

Supplementary Information

## Figures and Tables

**Table 1:** Mean accuracy at withholding response during unimodal auditory catch trials and responding to experimental trials (1- percent omission).

Experiment #		Catch Trials		Experimental Trials	
Experiment 1	**Condition**	**Correct Withholding (%)**	**S.E.M (%)**	**1-omission (%)**	**S.E.M (%)**
	Looming	98.10%	3.00%	97.40%	1.45%
	Receding	98.50%	2.80%	97.86%	1.35%
Experiment 2	Looming	99.50%	1.30%	98.40%	1.51%
	Receding	99.32%	2.20%	99.34%	1.27%
Experiment 3	Peri-Hand	97.22%	2.01%	97.46%	2.31%
	Peri-Trunk	98.10%	1.80%	98.21%	1.51%
Experiment 4	Peri-Hand Near	98.45%	0.90%	99.21%	1.17%
	Peri-Hand Far	99.10%	1.00%	99.31%	1.83%
Experiment 5	Peri-Face	99.20%	1.20%	98.70%	1.12%
	Peri-Trunk	98.80%	1.50%	98.68%	0.94%
Experiment 6	Peri-Face	99.13%	1.23%	98.10%	1.61%
	Peri-Trunk	99.14%	1.10%	98.45%	1.60%
Experiment 7	Looming	100%	0.00%	99%	0.64%
	Receding	99.20%	0.40%	98.62%	0.40%

Overall participants were very accurate, and therefore analysis focused on the RT data.
