# Supplementary material for: Body part-centered and full body-centered peripersonal space representations
Source: Sci Rep. 2015 Dec 22;5:18603. doi: 10.1038/srep18603 (PMC4686995; doi:10.1038/srep18603)
Supplement: Supplementary Information [file srep18603-s1.doc]

**SUPPLEMENTARY INFORMATION;**

**Body part-centered and full body-centered peripersonal space representations**

**Andrea Serino^1, 2, *^, Jean-Paul Noel^1, 2^, Giulia Galli^1, 2, 3^, Elisa Canzoneri^1, 2^, Patrick Marmaroli^4^, Hervé Lissek^4^, & Olaf Blanke^1, 2, 5^**

^1^ Laboratory of Cognitive Neuroscience, Brain Mind Institute, School of Life Sciences, Ecole Polytechnique Federale de Lausanne, Lausanne, Switzerland.

^2^ Center for Neuroprosthetics, School of Life Sciences, Ecole Polytechnique Federale de Lausanne, Lausanne, Switzerland.

^3^ Department of Psychology, Sapienza University, Rome, Italy.

^4^ Laboratory of Electromagnectics and Acoustics, Institute of Electrical Engineering, School of Engineering, Ecole Polytechnique Federale de Lausanne, Lausanne, Switzerland.

^5^ Department of Neurology, University Hospital, Geneva, Switzerland.

Corresponding Author:

Andrea Serino

[Andrea.Serino@epfl.ch](mailto:Andrea.Serino@epfl.ch)

+41 21 69 30695

Station 19, CH-1015 Lausanne

**2-speakers setup**

This setup was a replication of that used by Canzoneri et al. ^1-5^, Sounds were generated by adjusting the relative intensity of two loudspeakers (M-Audio studiophile AV 360, inMusic Brands Inc, Cumberland, RI) placed facing each other at a distance of 1 meter. One speaker was placed close to the participant (within ~ 5 cm), while the other one was placed far (~ 1 m) from the subject. Auditory stimuli were samples of pink-noise at 44.1 kHz. Sound intensity was manipulated through SOUNDFORGE 4.5 software (Sonic Foundry, Madison, WI) so that the intensity of looming sounds rose exponentially from 55 to 70 dB Sound Pressure Level (SPL), while the intensity of receding sounds decreased exponentially from 70 to 55 dB. Both loudspeakers were always co-activated. During the looming sounds the far loudspeaker was activated maximally at the beginning of the trial, and then its intensity decreased. The close loudspeaker adopted the opposite pattern of activation (minimum at first and then rose exponentially). As for the receding sounds, close and far loudspeakers reversed their patterns in comparison to the looming sound situation.

**Validation 2-speakers setup**

***Looming vs. Static***

In order to confirm that the dynamic sounds were perceived as moving in space and that they were perceived at different distances from the body at different onset delays, 14 blindfolded participants were presented with either the looming sound or a control static sound (both near and far speakers emitting pink noise at 70 dB) and were asked to orally indicate on a scale from 0 to 100 (near to far), where was the sound located when a vibrotactile stimulation was administered. Vibrotactile stimulation was administered at 10 different temporal delays from sound onset, so that 10 sound locations were probed. A 2 (movement: looming vs. static) x 10 (sound distances) Within-Subjects Analysis of Variance showed that there was no movement main effect (F(1,13) < 1, ns), nonetheless there was a significant main effect for distance (F(9, 117) = 32.940, p < 0.001, partial η^2^ = 0.71), and most importantly a movement X distance interaction (F(9, 117) = 17.779, p < 0.001, partial η^2^ = 0.591). Subsequent separate ANOVA’s showed that there was no main effect of distance (F(9, 117) = 1.182, p = 0.313) for the static sound, while a significant distance effect was present for the looming sound (F(9, 117) = 129.993, p < 0.001, partial η^2^ = 0.909) (Figure 1 , upper panel).

***Looming vs. Receding***

Similarly, in order to confirm that dynamic looming and receding sounds were indeed perceived as traveling in opposite direction, 6 blindfolded participants were presented with either a looming or a receding sound (as detailed above), either in the back-space or in the front-space, and were asked to orally indicate on a scale from 0 to 100 (near to far), where was the sound located when a vibrotactile stimulation was administered. Vibrotactile stimulation was administered at 6 different temporal delays from sound onset. A 2 (sound direction: looming vs. receding) x 2 (mapping space: back-space vs. front-space) x 6 (sound distances) Within-Subjects Analysis of Variance showed a significant main effect of distance (F(5, 25) = 11.367, p < 0.001, partial η^2^ = 0.695), while the rest of comparisons were non-significant (all p > 0.11). Importantly, the main effect of distance was independently present both for looming (F(5, 25) = 11.575, p < 0.001, partial η^2^ = 0.698) and receding (F(5, 25) = 9.406, p < 0.001, partial η^2^ = 0.653) sounds equally, as evidenced by the lack of distance X sound direction interaction (F(5, 25) = 0.653, p = 0.662).

**16-speakers setup**

Details concerning the algorithm governing the generation of sound sources with the 16-speakers setup can be found in the appendix below. In short, the rendering system was composed of two longitudinal arrays of eight loudspeakers each. These arrays were placed on either side of the participants. A broadband sound source was played simultaneously through all speakers while modulating via a Gaussian function the amplitude at each specific speaker in a time dependent-manner.

***Validation 16-speakers setup***

As for the case of the 2-speaker setup, we ran a sound localization experiment in order to demonstrate that the sound source generated by the 16-speaker setup gave the impression of a sound dynamically moving, which could be localized at a different distances from the participant’s body depending of the delay from the sound onset. Seven blindfolded participants, placed at the midpoint of the loudspeakers array, were first presented with static sounds originating from 1 or 2 meters in front of them, as well as 1 or 2 meters behind them. They were told that these sounds corresponded respectively to scores of 100, 200, -100, and -200. Next, participants were presented with sound looming toward them at a speed of 35cm/s, originating from either 1 meter in front of them and terminating 1 meter behind them, or originating 2 meters in front of them and terminating 2 meters behind them. At each out of 12 possible delays from sound onset, they were given a vibrotactile stimulation and they were asked to score on a scale from -200 to 200 where was the sound located at the moment of touch. In this way, 12 sound distances were sampled; six in the front and six in the back.

A 2 (Sound Starting point: +1 meter vs. +2 meters) x 12 (Sound Distance) Within-Subjects Analysis of Variance was carried out. Results exposed a Sound Distance main effect (F(11,66) = 17.765, p < 0.001, partial η^2^ = 0.748) and a Sound Starting point X Sound Distance interaction (F(11, 66) = 8.703, p < 0.001, partial η^2^ = 0.592). As Figure 1 (lower panel) shows, and as expected, when sounds approached in the front space, subjects clearly perceived auditory stimuli as presented closer to them at increasing temporal delays; vice-versa, when sounds receded in the back space, participants perceived the sound sources farther from their body, at increasing temporal delays. The Starting point X Sound Distance interaction was driven by the steeper slope for the sound originating from 2 meters away (Starting point: M = 190.35 cm, SD = 40.41 cm) and finishing two meters in the back (End point: M = -179.40 cm, SD = 44.74 cm), than for the sounds originating 1 meter in front (Starting point: M = 119.23 cm, SD = 27.45 cm) and terminating 1 meter in the back (End point: M = -113.57 cm, SD = 24.09 cm). In this manner, these results demonstrate both a sensitivity to sound distance, and as for the 2-speaker setup, a lack of differential sound localization ability for looming and receding sounds.

**Appendix**

**Hardware**

The audio rendering system was made of two uniform linear arrays (ULA) of eight loudspeakers each (JBL Control 1 Pro WH Pair), a USB audio interface with eight synchronous analog outputs (M-Audio FastTrack Ultra 8R), and two four-channel amplifiers feeding the speakers (Ecler MPA 4 80R). The two ULA’s were placed on the right and left sides of the participants as depicted in Figure S1. The distance $d$between two consecutive loudspeakers was set to 27.5 cm, and the distance $D$ between the two line arrays was set to 1 meter. The loudspeakers were fixed on two independent metallic structures that allow for horizontal and vertical positioning of the ULA’s.


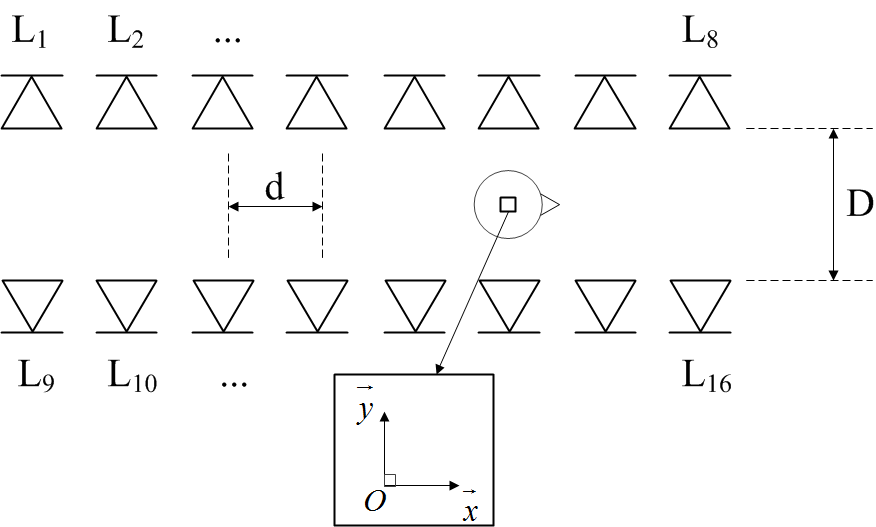


Figure S1: Schematization of the rendering system

**Stimulus synthesis**

The stimulus generated by the loudspeakers spatialized a moving broadband sound source moving through the participant from back to front, or front to back, at a constant speed. The global strategy adopted consisted in synchronously sending the same signal to all the speakers, while modulating the amplitude of each channel separately in order to give the subject the impression that the sound source was approaching, receding, or approaching and then receding, from and to a particular direction. The modulation applied on each channel was a function of i) the relative position of the Virtual Sound Source (VSS) from the participant, and ii) the relative position of the considered loudspeaker to the Participant Head Center (PHC).

Now, let us consider the PHC as being the center of an orthonormal basis $\mathbf{R}(O,\vec{x},\vec{y})$as shown in Figure S1. On this basis, let us denote the VSS position as $(x^{vss},y^{vss})$, and the position of the $q^{th}$ loudspeaker,$q\in\{1,2,...,16\}$, as $(x_{q}^{ls},y_{q}^{ls})$. Let us also assume that the VSS is forced to follow the x-axis direction only such that $y^{vss}=0$ at any time of the experiment. After Figure S1., it is clear that the loudspeakers can be grouped by pairs where a pair is composed of two loudspeakers with same abscissa. It follows, thus;

| $\begin{matrix} \text{pair} p=1 \text{ (loudspeaker 1 and 9)} \text{:} \left\{x_{1}^{ls} \text{=} x_{9}^{ls} , y_{1}^{ls} \text{=} -y_{9}^{ls}\right\} \\ \text{pair} p=2 \text{ (loudspeaker 2 and 10): }\left\{x_{2}^{ls} \text{=} x_{10}^{ls} , y_{2}^{ls} \text{=} -y_{10}^{ls}\right\} \\ ... \\ \text{pair} p=8 \text{ (loudspeaker 8 and 16): }\left\{x_{8}^{ls} \text{=} x_{16}^{ls} , y_{8}^{ls} \text{=} -y_{16}^{ls}\right\} \end{matrix}$ | (1) |
| --- | --- |

Since the objective was to give the impression of a sound source approaching toward or receding from the participant, the signal sent to a particular loudspeaker was the same as the signal sent to its facing loudspeaker. In practice this is achieved by connecting in series the two loudspeakers belonging to the same pair.

**Synthesis Algorithm**

The first step consisted in creating a pink noise of length $N$(in samples) and to duplicate it eight times so that each pair of loudspeaker could play its own signal. Let $s_{p}[n]$ be the signal vector of the$p^{th}$pair,
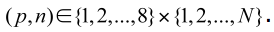
The integer $N$ is defined by the VSS speed $v^{vss}$ (in m/s). The VSS’ start and stop coordinates, $x_{start}^{vss}$ and $x_{stop}^{vss}$ (in meters) and the sampling frequency $f_{s}$ of the audio sound card (in Hz) are defined according to the following formula:

| $N=\left\lfloor\sqrt{\left(x_{stop}^{so}-x_{start}^{so}\right)^{2}}\times\frac{f_{s}}{v^{so}}\right\rfloor$ | (2) |
| --- | --- |

where stands for the floor function. In practice, we set $f_{s} = 44.1\text{ kHz}$, while , and varied according to the experiment (see below for details).

The second step consisted in dividing each signal vector into $M$frames of length $N_{f}$ (in samples) with an overlap of $N_{o}$ (in samples). This so-called framing procedure allowed updating the amplitude not every sample (i.e. every 0.02 ms), which is a time consuming computation, but every few ms only. In practice, we set $N_{f}=2048$ samples and$N_{o} =1024$ samples allowing an update of the signal amplitude every 23.22 ms. The number of frames$M$contained in a signal of length $N$ is given by the formula:

| $M=\left\lfloor\frac{N-N_{f}}{N_{f}-N_{o}}\right\rfloor+1$ | (3) |
| --- | --- |

Third, each frame was multiplied by a weighting factor$a_{mp}$, $m\in\{1,2,...,M\}$, whose role was to mimic the amplitude variation of a real moving sound source as a function of time and space. Here we set:

| $a_{mp}=a\times10^{\frac{60log_{10}(1/\lambda_{mp})}{20}}$ | (4) |
| --- | --- |

Where $a$ is a scalar allowing the operator to adjust the maximal sound pressure level at the ear of the subject (and play the same role as the gain variator of the amplifier) and$\lambda_{mp}$ is the distance (in meters) between the VSS and the $q^{th}$ loudspeaker at the instant $m$. This distance is defined by:

| $\lambda_{mp}\text{= }\sqrt{\left(x_{p}^{ls}-x_{m}^{vss}\right)^{2}+\left(\frac{D}{2}\right)^{2}}$ | (5) |
| --- | --- |

Note that, according to equation (4), the VSS pressure level decreases 60 dB per distance doubling. This value was chosen in order to mimic a realistic moving sound source. In theory, an omnidirectional sound source level decreases by only 6 dB^6^, however, setting this value does not give a realistic perception of moving sound, since we are not used to hear real omnidirectional broad band moving sound sources in nature.

The fourth step consisted in windowing each frame by a suitable window in order to avoid clicks when passing from one frame to the other. We chose the Hann window^7^ defined by:

| $w[\mathrm{k}]= 0.5\left[1-\left(\cos\frac{2\pi k}{N_{f}-1}\right)\right], \forall k\in\left\{1,2,...,N_{f}\right\}$ | (6) |
| --- | --- |

Finally, the eight final signals $\tilde{s}_{p}[n]$, were reconstructed by concatenating all the scaled and windowed frames according to Algorithm 1, described below.

$$\begin{matrix} \text{For} p=1 \text{to} 8 \text{(loop on channels)} \\ \text{For} m=1 \text{to} M \text{(loop on frames)} \\ j \leftarrow\left(m-1\right)\left(N_{f}-N_{o}\right) \\ \\ \text{For} k=1 \text{to} N_{f} \text{(loop on samples)} \\ \tilde{s}_{p}\left[j+k\right] \leftarrow\tilde{s}_{p}[\mathrm{j}+k] +a_{mp}w[\mathrm{k}]s_{p} [\mathrm{j}+\mathrm{k}] \\ \text{endfor} k \\ \\ \text{endfor} m \\ \text{endfor} p \end{matrix}$$

Algorithm 1: concatenation of the VSS signal

A schematic summary of the steps explained above in order to realistically simulate a moving source of sound through space and in depth is provided in Figure S2.


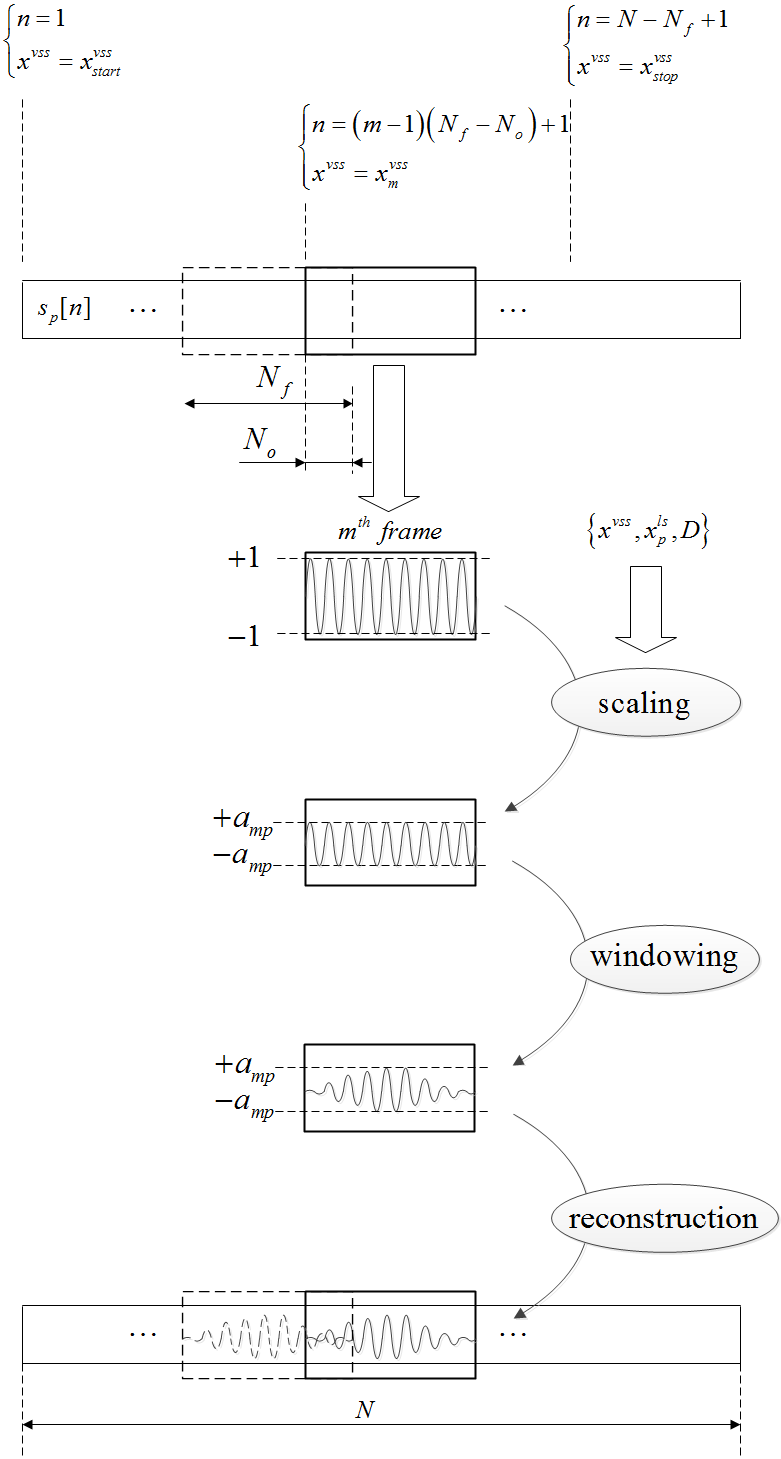


Figure S2: Schematization of the acoustic stimuli rendering procedure

**Supplementary References**

1. Canzoneri E, Magosso E, Serino A. Dynamic sounds capture the boundaries of peripersonal space representation in humans. PLoS One 7:e44306, 2012.

2. Canzoneri E, Marzolla M, Amoresano A, Verni G, Serino A. Amputation and prosthesis implantation shape body and peripersonal space representations. Sci Rep 3;3:2844, 2013a.

3. Canzoneri E, Ubaldi S, Rastelli V, Finisguerra A, Bassolino M, Serino A. Tool-use reshapes the boundaries of body and peripersonal space representations. Exp Brain Res. 228, 25–42, 2013b.

4. Bassolino M, Serino A, Ubaldi S, Làdavas E. Everyday use of the computer mouse extends peripersonal space representation. Neuropsychologia 48(3):803–811, 2010.

5. Teneggi, C., Canzoneri, E., di Pellegrino, G., & Serino, A. Social modulation of peripersonal space boundaries. Current biology: 23(5), 406–411, 2013.

6. Piercy, J.,E., & Daigle, G.,A. (1998). Acoustical measurement and noise control. Acoustical Society of America.

7. Salivahanan, S., Vallavaraj, A., & Gnanapriya, C. (2007) Digital signal processing. Tata McGraw-HIll Education.
